# Sperm mitochondrial DNA copy number: a marker of male fertility and reproductive success

**DOI:** 10.1093/humupd/dmag013

**Published:** 2026-05-22

**Authors:** Savni Sawant, Jeffrey M Mann, J Richard Pilsner

**Affiliations:** Department of Biochemistry, Microbiology, Immunology, School of Medicine, Wayne State University, Detroit, MI, USA; C.S. Mott Center for Human Growth and Development, Department of Obstetrics and Gynecology, School of Medicine, Wayne State University, Detroit, MI, USA; C.S. Mott Center for Human Growth and Development, Department of Obstetrics and Gynecology, School of Medicine, Wayne State University, Detroit, MI, USA; C.S. Mott Center for Human Growth and Development, Department of Obstetrics and Gynecology, School of Medicine, Wayne State University, Detroit, MI, USA; Institute of Environmental Health Sciences, Wayne State University, Detroit, MI, USA

**Keywords:** sperm mitochondrial DNA copy number, male infertility, spermatogenesis, TFAM, mtDNA dysfunction

## Abstract

**BACKGROUND:**

Sperm mitochondrial DNA copy number (mtDNAcn) has emerged as a promising biomarker of sperm health, providing molecular insight beyond what is captured by standard semen analysis. Elevated sperm mtDNAcn has been consistently associated with lower sperm motility, concentration, and morphology, as well as prolonged time-to-pregnancy in natural conception and reduced fertilization potential in ART.

**OBJECTIVE AND RATIONALE:**

This review summarizes current evidence on the biological underpinnings of sperm mtDNAcn, including its regulation during spermatogenesis, the role of nuclear-encoded mitochondrial proteins such as TFAM (*mitochondrial transcription factor A*), and its potential epigenetic modulation through sperm DNA methylation. We evaluate general population and clinic-based studies linking sperm mtDNAcn to semen quality, couple fecundity, and early embryo development, while highlighting methodological considerations such as quantification techniques and somatic cell contamination.

**SEARCH METHODS:**

A literature search was conducted to identify human studies evaluating sperm mtDNAcn in relation to male fertility, semen quality, reproductive outcomes, and embryology outcomes, as well as experimental models investigating the underlying biological mechanisms of mtDNA regulation during spermatogenesis via TFAM up to 1 April 2026. Searches were performed in PubMed, Web of Science, and Scopus using combinations of keywords and Medical Subject Headings (MeSH), including *sperm mitochondrial DNA copy number*, *mtDNAcn*, *male infertility*, *pregnancy outcomes, ART outcomes, semen quality*, *sperm epigenetics, TFAM, mitochondrial transcription factor A*, and sperm *mtDNA regulation*. Reference lists of relevant reviews and primary articles were manually screened to identify additional studies. Eligible studies included observational epidemiologic studies, clinical cohort studies, and experimental investigations that quantified sperm mtDNAcn and examined associations with semen parameters, fertility outcomes, or sperm epigenetics. No restrictions were placed on geographic location, while only articles published in English were considered.

**OUTCOMES:**

Across 21 epidemiologic, experimental, and clinical studies, elevated sperm mtDNAcn has been consistently associated with reduced sperm quality and inconsistently associated with diminished couple-level reproductive potential. Higher mtDNAcn is associated with lower sperm concentration, total sperm count, motility, and normal morphology, as well as higher sperm DNA fragmentation and chromatin abnormalities. It has also been linked to reduced likelihood of pregnancy and poorer embryo quality. Emerging evidence indicates associations between sperm mtDNAcn and altered nuclear DNA methylation patterns, supporting a role for mitochondrial–nuclear crosstalk. Collectively, these findings position sperm mtDNAcn as a biologically informative and clinically relevant indicator of male reproductive health that may complement, or potentially enhance, traditional semen analysis in both research and clinical settings.

**WIDER IMPLICATIONS:**

Sperm mtDNAcn holds promise as a biomarker for male fertility assessment, yet its full clinical potential has not been yet to be realized. Establishing standardized measurement protocols across sperm fractions and laboratory platforms will be essential for enabling cross-study comparability and facilitating clinical translation. Large prospective studies are needed to define clinically meaningful thresholds and better characterize the relationship between mtDNAcn alterations and spermatogenic impairment. Intervention strategies targeting mtDNA biogenesis and depletion, including antioxidant strategies and mitochondria-directed pharmacotherapy, warrant further investigation in the context of male fertility and fecundity. Collectively, sperm mtDNAcn may serve as an adjunctive marker in the assessment of male reproductive health and may inform future precision medicine approaches.

**REGISTRATION NUMBER:**

N/A.

## Graphical Abstract

**Graphical Abstract:**
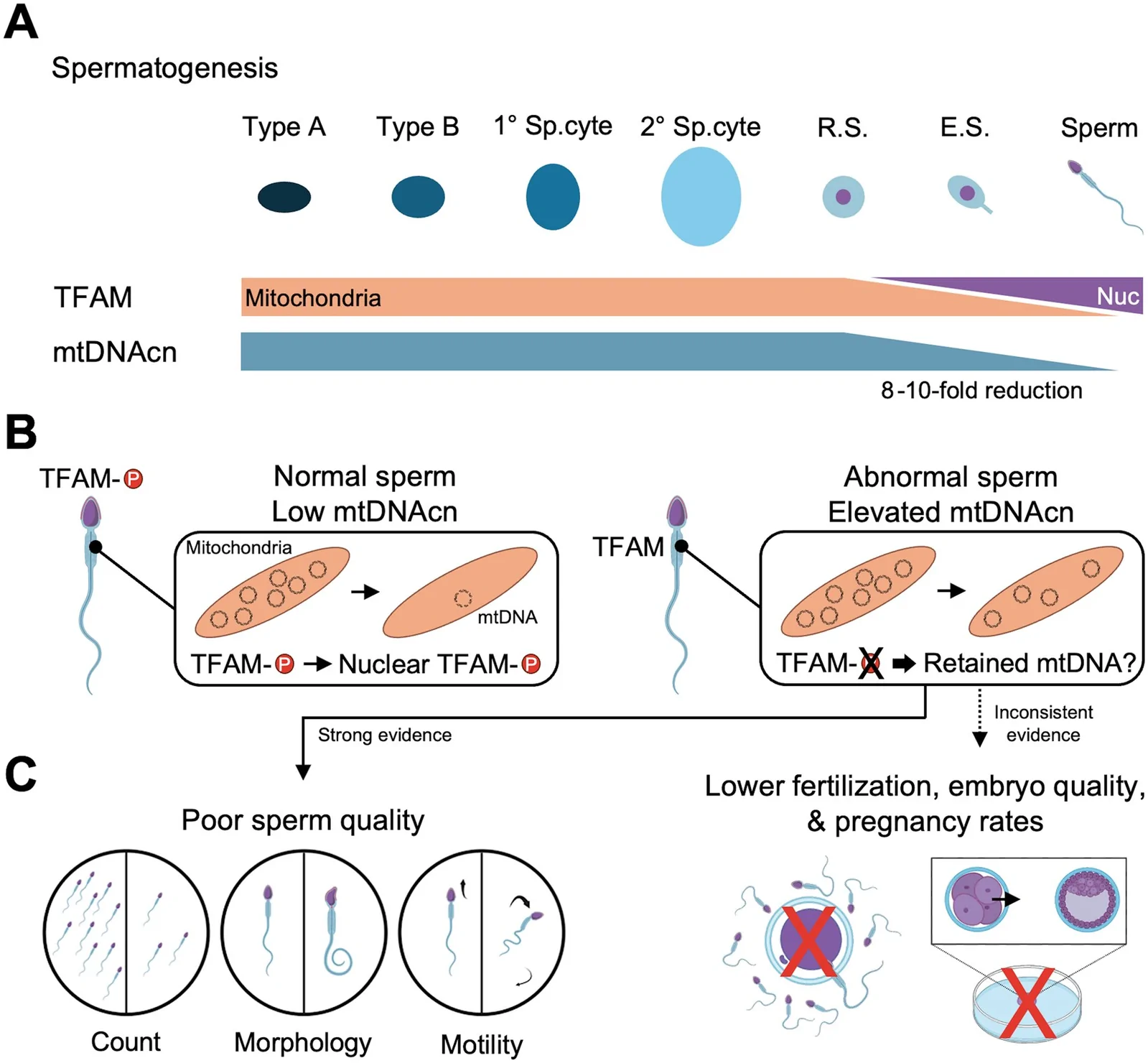
**Overview of depletion of human sperm mtDNA copy number during spermatogenesis and its implications for reproductive outcomes.** (A-B) Overview of depletion of human sperm mtDNA copy number during spermatogenesis and (C) its implications for reproductive outcomes. Abbreviations: Type A, type A spermatogonia; type B, Type B spermatogonia; 1° Sp.cyte, primary spermatocyte; 2° Sp.cyte, secondary spermatocyte; R.S., round spermatid; E.S., elongating spermatid; mtDNA copy number, mtDNAcn; TFAM, Transcription Factor A, Mitochondrial; Nuc, nucleus; P, phosphate. Created in BioRender. Sawant, S. (2026) https://BioRender.com/2uv2rqi.

## Introduction

Infertility is the inability of a couple to conceive after 1 year of unprotected intercourse, with male factor infertility solely responsible for 33% of cases (Agarwal *et al.*, 2021; WHO, 2023). Multiple factors such as genetic, occupational, or environmental exposures can cause male infertility and lead to dysfunction in both sperm production and function (Agarwal *et al.*, 2021). Fertility problems are further exacerbated by lifestyle factors like obesity and smoking and pose significant emotional, financial, and societal challenges. The prevalence of male infertility highlights the need for earlier identification of potential infertility in males, thus reinforcing the development of robust biomarkers for fertility assessment at the clinical level (Fovargue *et al.*, 2025).

Traditional semen analysis, which evaluates semen volume, sperm count, concentration, motility, and morphology, has been the standard method for assessing male fertility for decades (Guzick *et al.*, 2001; Jungwirth *et al.*, 2012; Bjorndahl *et al.*, 2022). However, these parameters have significant limitations. While they reflect overall sperm health, they do not fully capture the underlying molecular and biological processes that contribute to fertility (Sawant *et al.*, 2024). For example, a male with an average semen analysis may experience infertility due to factors such as elevated sperm DNA damage, increased oxidative stress, mitochondrial dysfunction, or mtDNA heteroplasmy, all of which are not reflected in standard semen testing, but can be assessed with advanced analysis (Sawant *et al.*, 2025). Moreover, semen parameters alone cannot explain idiopathic or unexplained subfertility among men with normal conventional semen parameters who are unable to conceive with their partners, underscoring the need to identify molecular biomarkers that provide deeper insight into male reproductive health and fertility while complementing existing semen analyses.

One such area for fertility research lies in the mitochondria, the energy-producing organelles in cells, which play a crucial role in sperm function and are critical for sperm motility during the fertilization process (Gray *et al.*, 2001). Sperm mitochondrial DNA copy number (mtDNAcn) has recently emerged as a promising biomarker for male fertility (May-Panloup *et al.*, 2003). Recent studies have demonstrated how high mtDNAcn in spermatozoa is often associated with poor semen quality and IVF outcomes, and an increased time to pregnancy for natural conception (Wu *et al.*, 2019a,b; Rosati *et al.*, 2020; Sawant *et al.*, 2025). These findings highlight the potential of mtDNAcn to serve as a complementary molecular biomarker beyond conventional semen analyses.

This review provides a comprehensive analysis of sperm mtDNAcn as a predictor of male fertility. We overview mitochondrial function in sperm and the impact of its dysfunction on fertility. Current research linking elevated mtDNAcn with poor semen quality, reduced embryo quality, and longer time-to-pregnancy is summarized, alongside novel molecular mechanisms of mtDNA elimination during spermatogenesis and potential factors influencing its retention. We examine molecular pathways involving nuclear-encoded mitochondrial genes and the relationship between mtDNAcn and sperm DNA methylation. The clinical utility of mtDNAcn in fertility diagnostics, particularly in ART, is discussed, and future research priorities include standardization of measurement protocols and therapeutic strategies to improve mitochondrial function.

## Methods

### Literature search strategy

A literature search was conducted to identify studies evaluating sperm mtDNAcn in relation to male fertility, semen quality, reproductive and embryological outcomes, and sperm epigenetics up to 1 April 2026. We also identified experimental studies investigating biological mechanisms relevant to sperm mtDNA regulation, including the role of mitochondrial transcription factor A (TFAM) during spermatogenesis. Searches were performed in PubMed, Web of Science, and Scopus using combinations of keywords and Medical Subject Headings (MeSH), including ‘sperm mitochondrial DNA copy number’, ‘mtDNAcn’, ‘male infertility’, ‘semen quality’, ‘semen parameters’, ‘reproductive outcomes’, ‘pregnancy outcomes’, ‘assisted reproductive technology (ART) outcomes’, ‘sperm epigenetics’, ‘TFAM’, ‘mitochondrial transcription factor A’, and ‘sperm mtDNA regulation’. Reference lists of relevant reviews, meta-analyses, and primary articles were also manually screened to identify additional eligible studies.

### Study eligibility

Studies were considered eligible if they were original research articles published in English and met at least one of the following criteria: (i) quantifying sperm mtDNAcn in human subjects and examining its association with the aforementioned parameters or outcomes; or (ii) were experimental studies relevant to biological mechanisms underlying sperm mtDNA regulation, particularly those involving TFAM and mitochondrial genome regulation during spermatogenesis or sperm development. Eligible human studies included observational epidemiologic studies, clinical cohort studies, case-control studies, and other original investigations directly assessing sperm mtDNAcn.

### Exclusion criteria

Studies were excluded if they did not directly quantify mtDNAcn in human sperm and instead relied on animal models (e.g. murine, bovine, porcine, or equine), or did not evaluate outcomes relevant to male fertility or reproduction. Specifically, we excluded studies focused on mtDNAcn measured in other cellular contexts, including blood, oocytes, embryos, or embryo culture media, unless they directly informed mechanistic questions related to sperm mtDNA regulation. Animal studies were excluded from the clinical and epidemiologic synthesis but were considered separately when providing mechanistic insight into TFAM biology and sperm mitochondrial DNA regulation. In addition, we excluded studies primarily focused on environmental, occupational, or pollutant exposures as determinants of sperm mtDNAcn where outcomes of semen quality, fertility, or reproductive outcomes were not assessed. Conference abstracts without full reports, non-English articles, and studies lacking sufficient methodological detail or original data were also excluded.

### Reporting of outcomes

Eligible studies were grouped according to the main outcome domain examined, including semen quality, male fertility or infertility status, ART and embryology outcomes, pregnancy or reproductive outcomes, sperm epigenetic outcomes, and mechanistic studies of mtDNA regulation during spermatogenesis. For each study, we summarized the study design, population, methods for quantifying sperm mtDNAcn, outcome definition, and the overall direction and significance of the reported findings. Given heterogeneity in study populations, laboratory methods, outcome definitions, and statistical approaches, findings were synthesized narratively rather than quantitatively. Outcomes were not pooled across studies, but were instead reported qualitatively by outcome category, direction of association, and overall consistency across studies.

## Overview of sperm mtDNA: function and quantification

Mitochondria are double membrane-bound organelles found in the cytoplasm of eukaryotic cells, often referred to as the ‘powerhouses’ of the cell (Javadov *et al.*, 2020). They are crucial for energy production through the process of oxidative phosphorylation, which generates ATP, the primary energy carrier in the cell (Glancy *et al.*, 2020). Structurally, mitochondria are characterized by an outer membrane and an inner membrane (Smeitink *et al.*, 2001; Protasoni and Zeviani, 2021). They are particularly crucial in high-energy-demand cells such as sperm, where mitochondrial integrity directly impacts motility and fertilization potential (Hirata *et al.*, 2002; Diez-Sanchez *et al.*, 2003; Moraes and Meyers, 2018; Durairajanayagam *et al.*, 2021).

**Figure 1.**
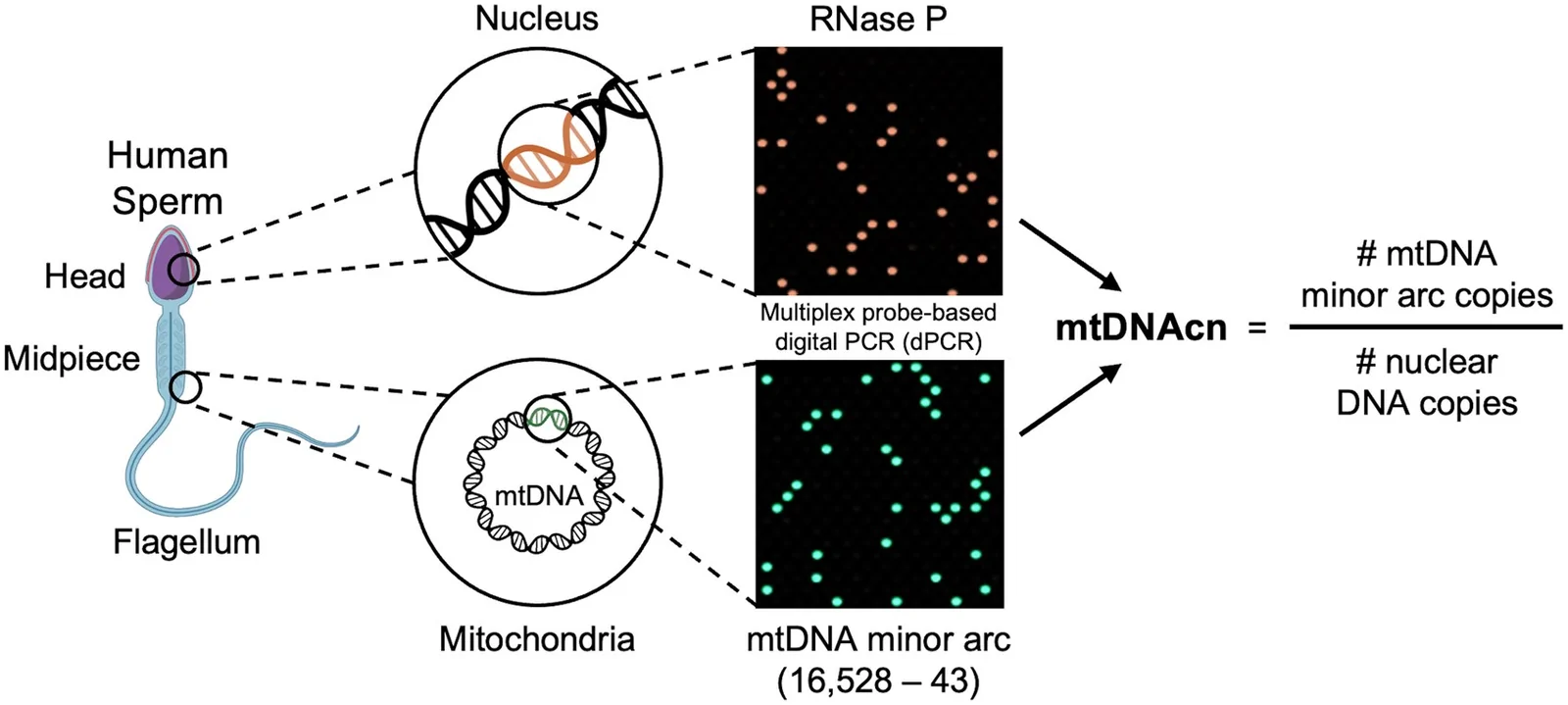
**Quantification of mtDNAcn via digital PCR (dPCR).** Schematic of multiplex dPCR analysis of human sperm targeting the minor arc of mtDNA and normalized to the single-copy nuclear DNA target, RNase P. mtDNA, mitochondrial DNA; dPCR, digital polymerase chain reaction; mtDNAcn, mitochondrial DNA copy number. Created in BioRender. Sawant, S. (2026) https://BioRender.com/jzpb6h0.

Unlike other non-nuclear eukaryotic organelles, mitochondria possess their own genetic material, mitochondrial DNA (mtDNA; Fig. 1), which is distinct from nuclear DNA (Allen and Coombs, 1980; Taanman, 1999; Hirata *et al.*, 2002). Structurally, mtDNA is a small, circular molecule ∼16 500 bp in length in humans, which is significantly shorter than the 3 billion base pairs of linear nuclear DNA. Despite its compact size, mtDNA encodes 13 essential proteins involved in oxidative phosphorylation, along with 22 transfer RNA genes and 2 ribosomal RNA genes (Taanman, 1999). Mitochondria in somatic cells can contain multiple copies of this circular DNA; however, in mature sperm, mitochondria are expected to harbor very few, if any, copies of mtDNA (Rantanen and Larsson, 2000).

In sperm, ATP is the primary energy source required for flagellar beating, which propels the sperm toward the oocyte. The mitochondria in sperm are located in the midpiece, a region at the base of the sperm head (which contains the nucleus), where they form tightly packed structures called the mitochondrial sheath, a helical structure around the central axoneme of the flagellum (Chen *et al.*, 2017). Mitochondria in sperm also help maintain cellular ion gradients, particularly calcium and potassium, which are essential for sperm activation. Furthermore, mitochondria in sperm closely regulate reactive oxygen species (ROS), which in low levels are necessary for sperm function and signaling, but in elevated levels can damage mitochondrial DNA and impair sperm motility (Chianese and Pierantoni, 2021). This highlights the delicate balance mitochondria must maintain to ensure sperm health and male fertility (Ruiz-Pesini *et al.*, 2000).

Unlike nuclear DNA, which is protected by multiple repair mechanisms, mitochondria have a limited capacity for DNA repair, making mtDNA more susceptible to mutations and therefore, dysfunction (Roubicek and Souza-Pinto, 2017). Furthermore, mitochondrial dysfunction is associated with poor semen quality, including abnormal sperm morphology, making it a significant factor in male infertility (Spiropoulos *et al.*, 2002). Consequently, sperm with damaged mitochondria may be less capable of navigating the female reproductive tract, leading to fertilization failure (Costa *et al.*, 2023).

Sperm mtDNAcn is most commonly quantified using quantitative PCR (qPCR), which calculates the relative ratio of mitochondrial to nuclear DNA by co-amplifying a mitochondrial target alongside a reference nuclear DNA target, offering high sensitivity, relatively low cost, and scalability for large sample sizes (Phillips *et al.*, 2014; Longchamps *et al.*, 2020; Bagdonaite *et al.*, 2025). Digital PCR (dPCR), a more recent advancement, leverages similar PCR assays but provides absolute copy number quantification by partitioning reactions into thousands of individual droplets, removing reliance on a standard curve and reducing inter-run variability, making it particularly advantageous for detecting subtle copy number differences in low-yield sperm DNA samples (Dong *et al.*, 2015; Shoop *et al.*, 2022). Whole genome sequencing approaches similarly derive mtDNAcn from the ratio of sequencing read depth of mitochondrial to nuclear DNA, while simultaneously capturing deletions and heteroplasmy for a more comprehensive mitochondrial profile, albeit at considerably greater cost and analytical complexity (Longchamps *et al.*, 2020). A key limitation shared across all methods is the absence of standardized reference genes, amplification targets, and normalization strategies, which may limit cross-study comparability (Guo *et al.*, 2009).

## Regulation of mtDNAcn during spermatogenesis

In humans and most eukaryotes, mtDNA is maternally inherited, and the mechanism of uniparental inheritance is well established (Chan and Schon, 2012; Luo *et al.*, 2018). Under this paradigm, only maternal mtDNA is transmitted to offspring, and this distinct mitochondrial genome can be clinically observed and evaluated in patients (Stewart and Larsson, 2014; Wei and Chinnery, 2020). Maternal-only inheritance is thought to be maintained to prevent mitochondrial genomic imbalance or the potential deleterious consequences of heteroplasmy during development, which can result in embryonic lethality or severe developmental defects (Wai *et al.*, 2010; El-Hattab *et al.*, 2017; Polovina *et al.*, 2020; Durairajanayagam *et al.*, 2021; Chen *et al.*, 2025). Although paternal mtDNA transmission has been reported in rare cases, this phenomenon remains controversial and is considered highly atypical (Chan and Schon, 2012; Mastrantonio *et al.*, 2019; Vertika *et al.*, 2020; Chen *et al.*, 2025). In sexual reproduction, the primary function of sperm is to deliver nuclear genetic material to the oocyte, thereby enabling transmission of the paternal genome to the next generation. During fertilization, sperm, including its midpiece mitochondria, which are typically devoid of mtDNA, penetrates the oocyte (Luo *et al.*, 2013, 2018). Since the oocyte contains the molecular machinery required to drive cell division and differentiation in the established zygote, paternal mitochondria are subsequently eliminated via mitophagy to preserve maternal mitochondrial and mtDNA inheritance (Sutovsky *et al.*, 2004; Rubio-Pena *et al.*, 2021; Sasaki and Sato, 2021). However, the disruption of this tightly regulated process, such as the persistence of mtDNA in sperm mitochondria, may compromise sperm function or interfere with fertilization and mitochondrial inheritance, thereby providing a biologically plausible link between elevated sperm mtDNAcn and reduced male fecundability.

The male germ cell lineage stems from primordial germ cells that originate in the epiblast during embryogenesis and give rise to spermatogonial stem cell populations required to maintain continuous sperm production (Ramalho-Santos *et al.*, 2009; Griswold, 2016; Neto *et al.*, 2016). In humans, spermatogenesis initiates during puberty, coinciding with hormonal changes that trigger the testes to produce sperm (Griswold, 2016; Neto *et al.*, 2016). Spermatogenesis encapsulates distinct phases in which germ cell populations must undergo mitotic and meiotic divisions, as well as morphological changes, to generate functionally mature sperm. Each of these events requires substantial energy, which are supplied via the mitochondria present within these germ cells (Ramalho-Santos *et al.*, 2009; Piomboni *et al.*, 2012; Moraes and Meyers, 2018; Vertika *et al.*, 2020; Boguenet *et al.*, 2021). Additionally, the structural changes occurring during spermiogenesis drive the redistribution of mitochondria within the cell (Yan *et al.*, 2019; Varuzhanyan and Chan, 2020; Boguenet *et al.*, 2021; Zhang *et al.*, 2022). The final localization of mitochondria resides in the midpiece of sperm, where they provide necessary energy to the flagellum for progressive motility, while the cytoplasm is effectively shed off via the cytoplasmic droplet and the nucleus condenses to form the sperm head (O’Donnell, 2014; Griswold, 2016; Neto *et al.*, 2016; Varuzhanyan and Chan, 2020; Zhang *et al.*, 2022). As spermatogenesis progresses, the number of mitochondria is reduced to ∼50–75 mitochondria per sperm, along with a concomitant 8- to 10-fold reduction in mtDNAcn, preparing sperm for ejaculation and fertilization (Hecht *et al.*, 1984; Rantanen and Larsson, 2000; Hirata *et al.*, 2002; Luo *et al.*, 2013; Moraes and Meyers, 2018; Yan *et al.*, 2019; Zlotorynski, 2023). The reduction of male germ cell mtDNAcn begins in post-meiotic haploid germ cells (round spermatids) prior to the onset of spermiogenesis and elongation (Chan and Schon, 2012; Luo *et al.*, 2013; Zlotorynski, 2023; Wang *et al.*, 2025). While the precise mechanisms underlying mtDNA clearance in human sperm are not fully understood, proposed pathways include ubiquitin-mediated processes or transcription factor-related regulation (Larsson *et al.*, 1997; Rajender *et al.*, 2010; Chan and Schon, 2012; Durairajanayagam *et al.*, 2021; Zlotorynski, 2023), or clearance via mitochondrial exonuclease activity analogous to that recently reported in fruit flies involving Poldip2 (Chen *et al.*, 2025). Disruption of these processes during spermatogenesis, resulting in elevated mtDNAcn in mature spermatozoa, has been associated with impaired male fertility. Accordingly, understanding the interactions between mtDNA and its associated proteins may provide key mechanistic insight into the regulated depletion of sperm mtDNAcn during spermatogenesis.

Proteins such as TFAM, POLG (DNA polymerase gamma), and TWNK (TWINKLE mtDNA helicase), all of which are nuclear encoded, play pivotal roles in mtDNA maintenance and function. POLG is the primary polymerase during mtDNA replication and repair and operates independently of nuclear DNA replication, while TWINKLE functions as the mtDNA helicase. Both proteins have been implicated in mtDNA elimination; however, the key to understanding the regulation of sperm mtDNAcn may lie with TFAM (Copeland and Longley, 2003; Tyynismaa *et al.*, 2004; Yu *et al.*, 2017; Remtulla *et al.*, 2019; Peter and Falkenberg, 2020). TFAM is a multifunctional protein that operates in mtDNA replication and transcription but also serves as the primary packaging molecule for mtDNA (Ekstrand *et al.*, 2004; Kang *et al.*, 2007; Lee *et al.*, 2014; Aasumets *et al.*, 2021; Bonekamp *et al.*, 2021; Kozhukhar and Alexeyev, 2023). In eukaryotic cells, mtDNA is packaged into nucleoids, which are nucleoprotein complexes where TFAM binds mtDNA through sequence-specific and non-sequence-specific interactions, effectively coating and compacting mtDNA copies within the matrix (Kang *et al.*, 2007; Yasukawa and Kang, 2023; Song *et al.*, 2024). These nucleoids contribute to mtDNA protection and transcriptional regulation, and as a result, may play a direct role in the regulation of mtDNAcn in sperm.

**Figure 2.**
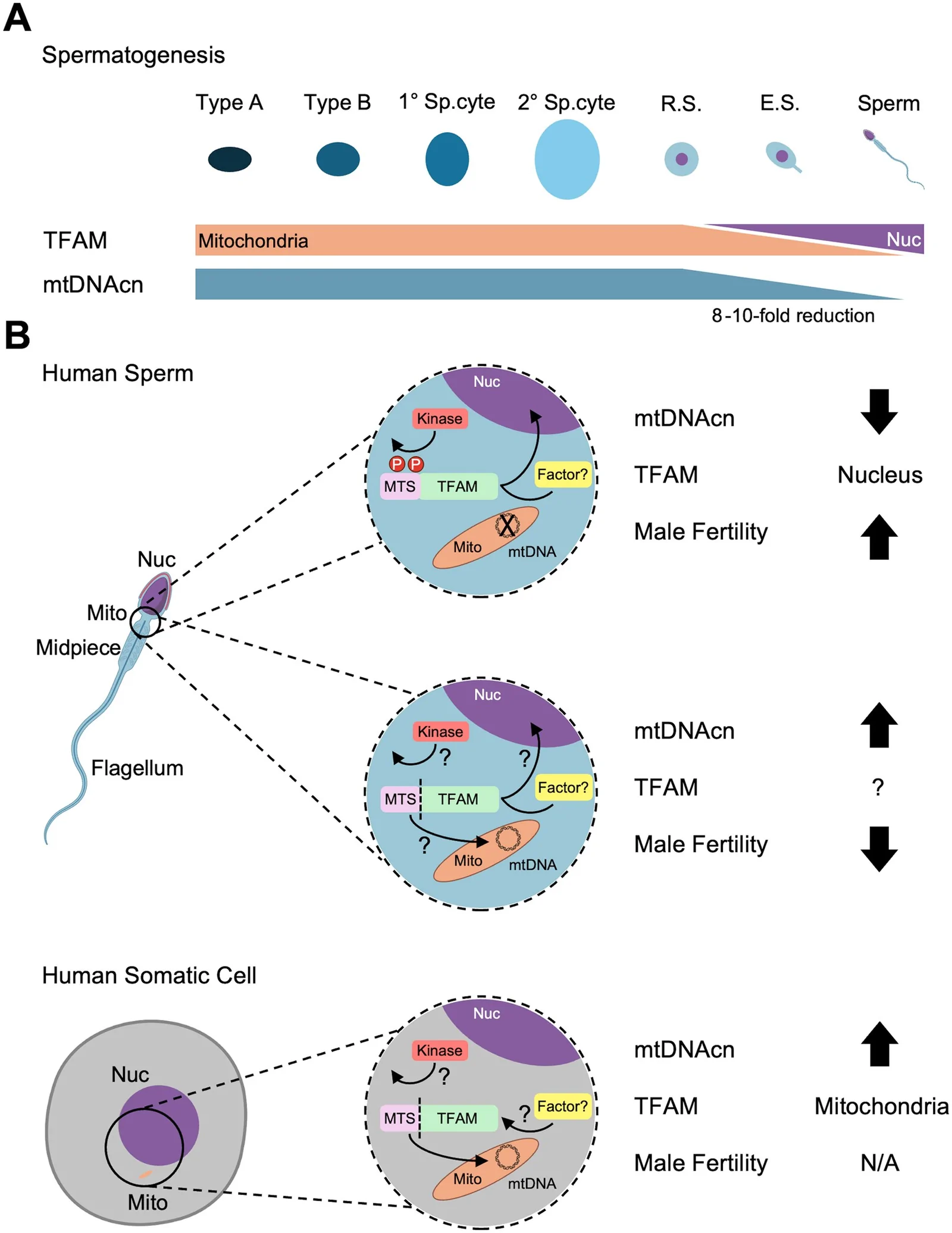
**Sperm mtDNAcn reduction during spermatogenesis is linked to TFAM localization.** (**A**) Mitochondrial DNA copy number (mtDNAcn) in male germ cells decreases as spermatogenesis progresses, with a pronounced reduction in post-meiotic germ cells, which coincides with TFAM delocalization from sperm mitochondria to nucleus. (**B**) Schematics of proposed mechanisms for mtDNA clearance via TFAM MTS phosphorylation status in human sperm and somatic cells, and their relationship to mtDNAcn, TFAM localization, and male fertility. TFAM, transcription factor A, mitochondrial; Type A, type A spermatogonia; type B, Type B spermatogonia; 1° Sp.cyte, primary spermatocyte; 2° Sp.cyte, secondary spermatocyte; R.S., round spermatid; E.S., elongating spermatid; Nuc, nucleus; Mito, mitochondria; P, phosphate. Created in BioRender. Sawant, S. (2026) https://BioRender.com/7f3aqbu.

In human somatic cells, TFAM has been shown to primarily exist within the mitochondria in an mtDNA-bound state (Kang *et al.*, 2007; Yasukawa and Kang, 2023; Liu *et al.*, 2024; Song *et al.*, 2024). However, TFAM in male germ cells behaves differently, reflecting the requirement to eliminate sperm mtDNA to preserve maternal mtDNA inheritance. As spermatogenesis progresses and the cytoplasm is shed during spermatid elongation, TFAM expression remains steady, yet importantly, its protein localization shifts from the mitochondria to the nucleus in the acrosome region of the sperm, while mitochondria remain confined to the midpiece (Bruser *et al.*, 2021; Lee *et al.*, 2023; Kavoosi *et al.*, 2024; Chen *et al.*, 2025). This relocalization occurs in post-meiotic male germ cells concomitantly with a reduction in mtDNAcn, suggesting that these two processes may be mechanistically linked (Fig. 2A) (Larsson *et al.*, 1997; Bruser *et al.*, 2021).

Building upon this discovery, Lee *et al.* (2023) further demonstrated that TFAM may be instrumental in sperm mtDNAcn reduction (Lee *et al.*, 2023). While TFAM localized to mitochondria in the cytoplasm in somatic cells, a sperm-specific isoform of TFAM was discovered in humans and non-human primates that was longer and localized to the nucleus (nuclear TFAM = 26 kD, mitochondrial TFAM = 25 kD) (Lee *et al.*, 2023; Zlotorynski, 2023). This longer isoform contains a mitochondrial targeting sequence (MTS) on the N-terminus and includes two serine residues (Ser31 and Ser34); phosphorylation of these residues prevents mitochondrial localization based on charge. This leads to sequestration of TFAM in the nucleus, although the exact mechanism underlying nuclear TFAM sequestration remains unclear and may require additional germ cell-specific factors that modify or chaperone TFAM (Fig. 2B).

Meanwhile, mitochondrial TFAM in somatic cells loses the MTS upon successful mitochondrial transport, resulting in the smaller TFAM isoform observed, which may reflect the absence of a corresponding kinase or additional regulatory factor, the identity of which remains unknown (Fig. 2B) (Lee *et al.*, 2023). Lee *et al.* (2023) showed that substitution of these key serine residues with alanine (S31A, S34A) prevented the phosphorylation of the MTS and caused TFAM in sperm to be retained in the midpiece, which may be consequential for mtDNA stability. Kinases PKA and ERK1/2 have been shown to phosphorylate TFAM; however, it is unclear which kinase is responsible for phosphorylation of these MTS residues of sperm TFAM *in vivo* (Lu *et al.*, 2013; Kang *et al.*, 2018). Altogether, this model proposed by Lee *et al.* is referred to as the ‘TFAM sequestration’ model, and represents one competing theory of mtDNA elimination in sperm. The alternative ‘mtDNA turnover’ model proposes that mtDNA replication factors are progressively reduced throughout spermatogenesis, allowing for gradual mtDNA loss over time (Lee *et al.*, 2023; Kozhukhar *et al.*, 2025).

**Figure 3.**
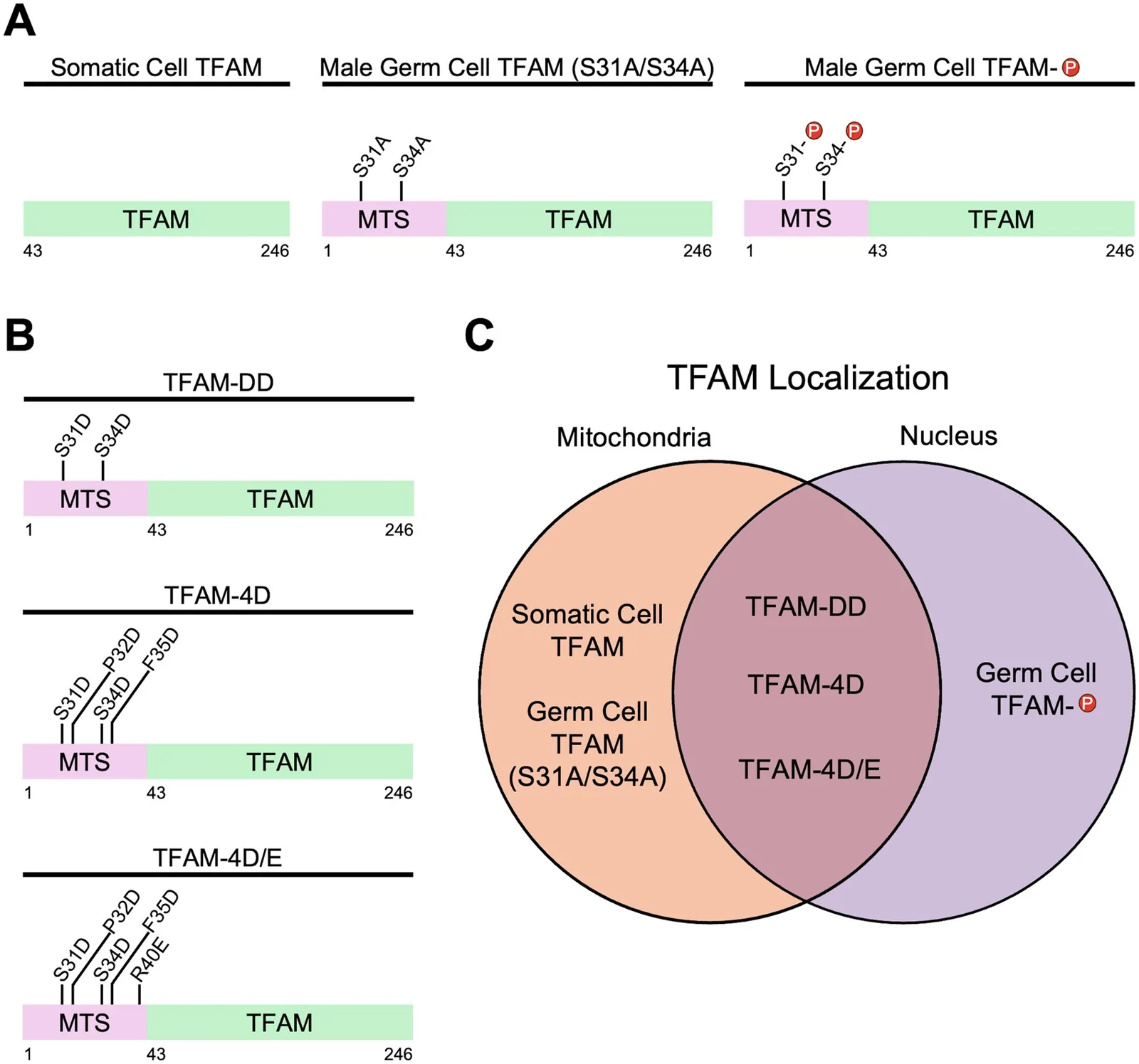
**TFAM MTS phosphorylation and charge do not solely dictate localization.** (**A**) Schematics of somatic cell and sperm isoforms of human TFAM emphasizing sperm-specific MTS and key amino acid residues. (**B**) Schematics of *in vitro* human TFAM phosphomimetic proteins with mutated key amino acid residues to alter charge and MTS cleavage. (**C**) Summary of organelle localization of TFAM proteins described in (A) and (B) through experiments performed by Lee *et al*. (2023) and Kozhukhar *et al*. (2025) (Kozhukhar and Alexeyev, 2023; Lee *et al*., 2023). TFAM, transcription factor A, mitochondrial; MTS, mitochondrial transport sequence; P, phosphate.

Interestingly, a recent report by Kozhukhar *et al.* (2025) suggested that phosphorylation of TFAM alone in sperm may not be the primary driver of TFAM mislocalization and mtDNA loss (Kozhukhar *et al.*, 2025). While Lee *et al.* identified sperm-specific TFAM isoforms with distinct intracellular localization based on MTS phosphorylation (Fig. 3A), Kozhukhar *et al.* (2025) proposed that these phosphorylation events, leading to MTS charge differences, did not by themselves drive TFAM mislocalization to the nucleus. To evaluate this hypothesis, they constructed *in vitro* TFAM phosphomimetic proteins mimicking phosphorylation of TFAM to differing degrees (TFAM-DD and TFAM-4D), including an isoform incapable of MTS cleavage (TFAM-4D/E) (Fig. 3B). They found that these TFAM phosphomimetic proteins did not exclusively localize to the nucleus (Fig. 3C), suggesting that TFAM mislocalization in sperm may not be dependent on MTS phosphorylation alone (Kozhukhar *et al.*, 2025). Collectively, these findings indicate that while TFAM may play a direct role in sperm mtDNA maintenance, additional regulatory factors are likely required to drive its relocalization, representing an important area for future investigation.

TFAM has been shown to directly interact with mtDNA *in vivo*; however, it remains unclear if mtDNA retention would result in TFAM mislocalization in infertility patients, including those with elevated sperm mtDNAcn. Thus, investigating TFAM as a potential biomarker for sperm mtDNAcn is warranted given the inverse relationship for TFAM nuclear localization and mtDNAcn reduction observed in humans (Lee *et al.*, 2023). While TFAM nuclear localization has been observed in human sperm, its relationship with varying levels of sperm mtDNAcn remains to be systematically evaluated (Lee *et al.*, 2023).

Additionally, direct visualization of sperm mtDNA retained within the midpiece could have profound clinical applications by complementing and enhancing traditional semen analysis and providing insight into the underlying molecular mechanisms not captured by conventional semen parameters. This may be achieved through visualization of TFAM localization in sperm of patients, given its direct interaction with mtDNA in somatic, or through interrogation of other molecular targets associated with mtDNA retention or TFAM mislocalization, including additional factors associated with sperm-specific TFAM. Ultimately, the ability to quickly and accurately identify elevated mtDNAcn in clinical sperm samples could improve the diagnosis of male infertility and uncover mechanistic understanding for patients with normal semen parameters yet unsuccessful conception.

## Sperm mtDNAcn as a biomarker of male fertility among infertility cohorts

In the past three decades, mtDNAcn in sperm has emerged as a promising molecular marker for evaluating male fertility, reflecting underlying mitochondrial function, oxidative stress, and cellular health beyond the limits of conventional semen analysis (Wu *et al.*, 2019a,b; Sun *et al.*, 2022). A growing body of evidence supports the clinical relevance of elevated sperm mtDNAcn as a feature of impaired sperm quality and subfertility (Zhang *et al.*, 2016; Aviles-Ramirez *et al.*, 2022). The following studies illustrate this relationship across various populations and methodological frameworks. Table 1 lists a summary of these findings.

**Table 1.** Summary of published studies examining sperm mtDNAcn and semen quality.

Study	Setting	Elevated sperm mtDNAcn was associated with	Method and targets
May-Panloup *et al.* (2003)	Infertility cohort	Abnormal semen group defined by at least one of: low sperm count, motility or morphology; more pronounced with two or more abnormal semen parameters	qPCR; mt: light strand D41 & R56 nuclear: beta-globin
Diez-Sanchez *et al.* (2003)	General population	Lower progressive motility; enriched in non-progressively motile/poorer-motility semen fraction compared to progressively motile fraction	Slot-blot ^32^P-labeling; mt: 16S rRNA nuclear: 18S rRNA
Song and Lewis (2008)	Infertility cohort	Abnormal semen group; lower sperm count	qPCR; mt: 16S rRNA nuclear: GAPDH
Tian *et al.* (2014)	Infertility cohort	Lower sperm concentration and motility (including CASA overall movement quality); lower morphology;	qPCR; mt: MTF3212 nuclear: beta-actin
Wu *et al.* (2019a)	Infertility cohort	Lower sperm concentration, total sperm count, motility, morphology	Multiplex qPCR; mt: minor-arc nuclear: RNaseP
Sun *et al.* (2022)	General population	Lower sperm concentration; total sperm count, total motility, and progressive motility	qPCR; mt: ND1 nuclear: beta-actin
Shi *et al.* (2022)	Infertility cohort	Abnormal semen defined by at least one below WHO cutoffs: concentration, total motility, or morphology	MultiplexqPCR; mt: minor-arc nuclear: RNaseP
Geng *et al.* (2024)	Infertility cohort	Asthenozoospermia; lower total motility in asthenzoospermic group but no relationship in the normozoospermic group	qPCR; mt: ND1 nuclear: beta-globin
Sawant *et al.* (2025)	General population	Reduced semen quality metrics of 32 basic and detailed semen parameters (measured with CASA), higher DFI and HDS	Multiplex dPCR; mt: minor-arc nuclear: RNaseP

mt, mitochondrial DNA PCR target; nuclear, nuclear DNA PCR target; qPCR, quantitative polymerase chain reaction; CASA, computer-assisted semen analysis; dPCR, digital polymerase chain reaction; DFI, DNA Fragmentation Index; HDS, high DNA stainability.

In one of the earliest investigations, May-Panloup *et al.* (2003) quantified sperm mtDNAcn using qPCR in a cohort of 67 men, including 32 with normozoospermia and 35 with abnormal semen profiles based on the WHO criteria at the time. The mean mtDNA/beta-globin ratio (mtDNAcn) was significantly higher in abnormal samples (6.1 ± 6.3) compared to normal samples (1.4 ± 0.9). Among samples with multiple morphological abnormalities, the mean mtDNAcn reached 9.1 ± 7.0. Additionally, fractionated sperm layers emphasized this trend, with low-density, representing poor quality, sperm had higher mean mtDNAcn compared to high-density sperm (17.1 ± 11.6 and 2.5 ± 2.5, respectively). Based on these findings, the authors suggested that elevated mtDNAcn was indicative of mitochondrial dysregulation in men with decreased semen quality, possibly reflecting a compensatory response to mitochondrial dysfunction (May-Panloup *et al.*, 2003).

A subsequent study by Song and Lewis (2008) evaluated mtDNAcn and mitochondrial integrity in sperm from 58 infertile men and 12 fertile controls. Their results reported that infertile men had significantly higher mtDNAcn (mean 34.0 copies/sperm, range 2.8–226) compared to fertile controls (mean 15.7 copies/sperm, range 3.2–48.5). Additionally, mtDNA deletions, which were assessed using qPCR targeting the common 4977-bp deletion, were more frequent in the infertile group than in the fertile control group (60% vs 8%). Notably, mtDNAcn was inversely correlated with sperm concentration (*R* = −0.28) and motility (*R* = −0.30), indicating that both mtDNA quantity and genomic integrity are associated with impaired sperm function (Song and Lewis, 2008).

The role of mtDNAcn in relation to both genetic and epigenetic sperm features was investigated by Tian *et al.* (2014), who examined 47 men attending an infertility clinic. Using qPCR for mtDNAcn and bisulfite sequencing to assess DNA methylation (LINE-1, H19, PEG3), they reported significant inverse correlations between mtDNAcn and sperm concentration (*R* = −0.44), motility (*R* = −0.43), and morphology (*R* = −0.31). Multivariable logistic regression models, adjusted for age, body mass index (BMI), abstinence duration, and lifestyle factors, revealed that men in the highest mtDNAcn tertile had a 4.9-fold increased odds of reduced motility (95% CI: 1.2–19.8). This study was one of the first to highlight potential mitochondrial-nuclear crosstalk underlying sperm dysfunction, as mtDNAcn was also associated with altered DNA methylation of key imprinted genes in sperm (Tian *et al.*, 2014).

In contrast to intracellular mtDNA, Chen *et al.* (2018) assessed cell-free mitochondrial DNA copy number (cf-mtDNAcn) in seminal plasma as an indirect marker of testicular and sperm health. Among 205 men undergoing fertility assessment, those with asthenozoospermia (n = 78) or oligoasthenozoospermia (n = 44) exhibited significantly lower cf-mtDNAcn compared with normozoospermic men (n = 83). Seminal cf-mtDNAcn was positively correlated with progressive motility (*R *= 0.67), total motility (*R *= 0.64), sperm concentration (*R *= 0.45), and normal morphology (*R *= 0.32). Conversely, cf-mtDNAcn was inversely associated with ROS as measured by the chemiluminescence assay (*R* = −0.57). The authors suggested that lower cf-mtDNAcn reflects increased oxidative stress and impaired mitochondrial turnover, further linking mtDNA-related metrics to functional sperm decline (Chen *et al.*, 2018).

More recently, in a prospective study of 119 men undergoing fertility treatment, Wu *et al.* (2019a) examined mtDNAcn via probe-based multiplex qPCR to assess associations with conventional semen parameters (Wu *et al.*, 2019a). In models adjusting for male age, higher mtDNAcn was significantly associated with reduced semen quality. Specifically, mtDNAcn was inversely associated with sperm concentration (β = −0.38), total sperm count (β = −0.35), progressive motility (β = −0.32), and normal morphology (β = −0.30). These associations remained significant even when accounting for abstinence time and other potential confounders. Importantly, the strongest signal emerged when infertility was defined by repeated measures: mtDNAcn showed high discriminatory ability for consecutive diagnoses of clinical infertility (Receiver operating characteristics with AUC = 0.91), which was comparable to key semen parameters (Wu *et al.*, 2019a,b). In a case–control study of 200 Iraqi men (150 infertile, 50 fertile controls), infertile men exhibited significantly higher sperm mtDNAcn and shorter sperm telomere length compared with controls, with the most pronounced changes observed in men with oligoasthenoteratozoospermia (Kadhim *et al.*, 2025). Across all participants, mtDNAcn and telomere length were inversely correlated (r = −0.56), and sperm mtDNAcn demonstrated stronger discriminatory performance for infertility status than telomere length (mtDNAcn AUC = 0.95 vs telomere length AUC = 0.89), with the difference being statistically significant (Kadhim *et al.*, 2025).

## Sperm mtDNAcn as a biomarker of male fertility in the general population

The relationship between mtDNAcn and semen quality was further investigated in a large cross-sectional study by Sun *et al.* (2022), which analyzed 5739 semen samples from 1164 potential sperm donors at the Hubei Province Human Sperm Bank in China. Using probe-based multiplex qPCR, the authors quantified sperm mtDNAcn and evaluated its association with key semen parameters. After adjusting for age, abstinence time, and other covariates, they reported that men in the highest quartile of mtDNAcn exhibited significantly lower progressive motility (–8.9%), total motility (–8.0%), sperm concentration (–42.8%), and total sperm count (–44.3%) compared with those in the lowest quartile. These associations remained significant in both linear and spline regression models, demonstrating a clear dose–response relationship between elevated mtDNAcn and impaired semen quality. The authors proposed that increased mtDNAcn likely reflects underlying mitochondrial dysregulation and compensatory mitochondrial biogenesis in response to subclinical sperm dysfunction, reinforcing the potential utility of mtDNAcn as a population-level biomarker of male reproductive health (Sun *et al.*, 2022).

Additional evidence from a general population setting was provided by Zhang *et al.* (2016) in the Male Reproductive Health in Chongqing College students (MARCHS) cohort study conducted in Chongqing, China (Zhang *et al.*, 2016). Among 656 young men, a subset of ∼386 participants underwent mitochondrial profiling, including assessment of sperm mtDNAcn, mitochondrial membrane potential (MMP), and mtDNA integrity. Sperm mtDNAcn was inversely associated with sperm concentration (*R* = −0.21), total sperm count (*R* = −0.23), and progressive motility (*R* = −0.16), all of which were statistically significant. In contrast, MMP and mtDNA integrity demonstrated positive correlations with semen volume, sperm concentration, total count, and motility. Multivariable regression models, adjusting for age, smoking, and occupational exposures, suggested that mitochondrial biomarkers were independent predictors of semen quality. This study was notable for demonstrating that the relationship between elevated mtDNAcn and impaired sperm function is evident not only in clinical infertility cohorts but also among healthy men from the general population, underscoring the broad applicability of mtDNAcn as a biomarker of male reproductive potential (Zhang *et al.*, 2016). In another general population setting from a preconception cohort, in a study by Sawant *et al.* (n = 281), sperm mtDNAcn (measured with probe-based dPCR) was broadly associated with semen quality: higher mtDNAcn significantly correlated with poorer conventional and detailed semen parameters (measured by CASA), and was inversely associated with a composite ranked sperm quality index (rho = −0.51). SCSA measures were also related to mtDNAcn, with higher mtDNAcn positively correlated with DNA fragmentation (DFI; rho = 0.20) and high DNA stainability (HDS; rho = 0.27), both meeting the Bonferroni-corrected significance threshold.

## Summary of sperm mtDNAcn and semen quality

In a recent meta-analysis, Popova *et al.* (2022) summarized evidence from 10 independent studies to evaluate differences in mtDNAcn between men with normal versus abnormal semen profiles. The pooled analysis included over 900 men and revealed a standardized mean difference of 1.08 (95% CI: 0.74–1.43) indicating significantly higher mtDNAcn in men with suboptimal semen parameters. Seven of the included studies also reported consistent inverse correlations between mtDNAcn and sperm count, motility, or morphology (mean *R* = −0.30). The authors rated methodological quality as ‘good’ or ‘very good’ in over 60% of studies included in the meta-analysis and concluded that sperm mtDNAcn is a reliable biomarker of male fertility impairment across diverse populations and study designs (Popova *et al.*, 2022). Taken together, these studies provide convergent evidence, drawn from diverse populations and methodological approaches, that elevated sperm mtDNAcn is a reproduceable marker of poor semen quality. Whether arising from compensatory mitochondrial biogenesis or increased oxidative stress, multiple studies suggest that higher mtDNAcn may reflect underlying mitochondrial dysfunction that compromises sperm quality. Alternatively, elevated mtDNAcn may indicate disruption of the tightly regulated depletion of mtDNAcn during spermatogenesis, potentially mediated through TFAM-dependent or other regulatory pathways discussed above, suggesting that retention of excess mtDNAcn in mature sperm reflects abnormal or incomplete spermatogenesis. Collectively, these findings support the utility of mtDNAcn as a non-invasive biomarker of male reproductive health, which may complement traditional semen analysis in diagnostic and prognostic frameworks (Huffman *et al.*, 2018; Rosati *et al.*, 2020; Sawant *et al.*, 2025).

## Sperm mtDNAcn in relation to reproductive outcomes

Building on the reproducible inverse relationships with mtDNAcn and conventional semen quality parameters detailed above, an important question is whether mtDNAcn provides clinically meaningful information beyond standard semen analysis. For sperm mtDNAcn to be considered a novel and useful biomarker, its relevance must extend beyond correlations with sperm concentration, motility, and morphology to demonstrate independent or incremental predictive value for reproductive outcomes. Its relationship must go beyond those of semen parameters by providing prediction of reproduction success outcomes. Specifically, biomarkers intended for clinical translation should inform key endpoints such as couple fecundity, fertilization, embryo development, and pregnancy outcomes, ideally outperforming or complementing existing semen parameters. The following section therefore examines the evidence linking sperm mtDNAcn with reproductive success in both natural conception and assisted reproductive settings, with particular attention to whether mtDNAcn adds prognostic insight beyond conventional measures of sperm quality. These findings are summarized in Table 2.

**Table 2.** Summary of published studies examining sperm mtDNAcn and embryology or reproductive outcomes.

Study	Setting	Elevated mtDNAcn was associated with	Method and targets
Wu *et al.* (2019b)	Infertility cohort	Lower fertilization rates; lower high quality Day 3 and transfer quality D5 embryos	Multiplex-qPCR; mt: minor-arc nuclear: RNaseP
Rosati *et al.* (2020)	General population	Lower fecundability and longer time-to-pregnancy	Multiplex-qPCR; mt: minor-arc nuclear: RNaseP
Tiegs *et al.* (2020)	Infertility cohort	No associations using ICSI for fertilization, blastocyst development, euploidy, pregnancy/live birth in adjusted models	TaqMan qPCR; mt: 16S rRNA nuclear: AluYB8 (rep seq)
Shi *et al.* (2022)	Infertility cohort	Lower fertilization rate in unadjusted analyses, effects attenuated with covariate adjustment; no association with downstream pregnancy outcomes post-embryo transfer	Multiplex qPCR; mt: minor-arc nuclear: RNaseP
Sawant *et al.* (2025)	General population	mtDNAcn improved predictive value of pregnancy when combined with semen parameters in machine-learning models	Multiplex dPCR; mt: minor-arc nuclear: RNaseP

mt, mitochondrial DNA PCR target; nuclear, nuclear DNA PCR target, qPCR, quantitative polymerase chain reaction, dPCR, digital polymerase chain reaction; rep seq, repetitive sequence.

Beyond semen quality, several studies have examined the role of mtDNAcn in determining couple fecundity and early embryo development. Wu *et al.* (2019), in one of the earliest investigations of mtDNAcn in a clinical fertility setting, examined the relationship between sperm mtDNAcn, via probe-based qPCR, and embryology outcomes in 119 men undergoing ART. Multivariable regression analyses showed that elevated mtDNAcn was significantly associated with reduced odds of successful fertilization (adjusted OR = 0.91 per 10-copy increase), high-quality embryos at cleavage stage (adjusted OR = 0.88) and transferable-quality embryos at the blastocyst stage (adjusted OR = 0.90). These associations remained statistically significant even after controlling for conventional semen parameters, suggesting that mtDNAcn burden contributes independently to early embryo developmental competence (Wu *et al.*, 2019b).

Moving beyond embryo development to pregnancy outcomes in women’s health, Ye *et al.* (2020) explored maternal mitochondrial biomarkers in chorionic villous samples from 75 women who experienced early pregnancy loss and 75 matched controls with ongoing pregnancies. The authors reported significantly higher mtDNAcn (log2-transformed mean: 3.08 vs 2.78) in the early pregnancy loss group. In multivariable logistic regression, each unit increase in mtDNAcn was associated with 71% higher odds of early pregnancy loss (adjusted OR = 1.71). These findings implicate mtDNAcn dysregulation as a contributing factor in early gestational failure, potentially originating from gametic or early embryonic dysfunction (Ye *et al.*, 2020). Similarly, elevated sperm mtDNAcn could also cause embryonic dysfunction, given that activation of the paternal genome occurs around Day 3 of embryogenesis.

Rosati *et al.* (2020) were among the first to evaluate mtDNAcn in the context of natural conception. In this prospective cohort of 384 couples from the general population attempting pregnancy, sperm mtDNAcn was quantified at baseline and time to pregnancy was tracked for over 12 months. Men in the highest tertile of mtDNAcn had a 50% reduction in the odds of conception in a given menstrual cycle (fecundability odds ratio = 0.50, 95% CI: 0.30–0.83) and an 18% lower cumulative probability of conceiving within 1 year (risk difference = −18.2%, 95% CI: −32.2%, −4.3%) compared to those in the lowest tertile. These associations remained significant after adjusting for male age, BMI, smoking, and other covariates. The results indicate that elevated sperm mtDNAcn may serve as a non-invasive biomarker of fecundability among couples in natural conception contexts (Rosati *et al.*, 2020).

To expand on these findings using predictive modeling approaches, Sawant *et al.* (2025) applied a machine learning framework using elastic net regression, integrating conventional semen parameters with sperm mtDNAcn to estimate couple fecundity. In a cohort of 281 couples attempting conception, model performance was evaluated for predicting time to pregnancy. Compared to individual semen measures, sperm mtDNAcn was the most predictive single parameter of pregnancy at 12 menstrual cycles in receiver operating characteristic (ROC) analyses (AUC, 0.68). Elastic net models combining predictive individual parameters into a weighted score demonstrated that the inclusion of mtDNAcn with conventional semen parameters improved model accuracy, with the area under the ROC curve increasing from 0.68 (95% CI: 0.62–0.74) to 0.76 (95% CI: 0.71–0.81). Additionally, sensitivity for identifying couples who would take longer than 12 months to conceive improved with the addition of mtDNAcn from 58% to 71% without compromising specificity. These results highlight the incremental predictive value of mitochondrial biomarkers in predicting reproductive potential, particularly when integrated with standard semen parameters (Sawant *et al.*, 2025).

Although the above-discussed studies show associations between sperm mtDNAcn and reproductive outcomes, results across investigations have not been entirely consistent. Tiegs *et al.* (2020) analyzed 2092 sperm samples from men undergoing IVF or ICSI. Using real-time PCR to quantify mtDNAcn, the authors evaluated associations with fertilization, blastocyst formation, embryo euploidy, and live birth. After adjusting for male pre-wash motility and female age, mtDNAcn was not found to be significantly associated with fertilization rate, usable blastocyst development, embryo euploidy, or live birth (Tiegs *et al.*, 2020). Despite being one of the largest studies to date, this study employed a non-standard approach to normalize sperm mtDNAcn to the nuclear genome. The preferred choice for nuclear reference for normalization is a single-copy gene; however, this study used the highly repetitive element, AluYB8. AluYB8 has previously been applied in single-cell analyses or whole genome amplification contexts to mitigate issues such as allele drop-out (Fragouli *et al.*, 2015). Given that AluYB8 sequences are present in the human genome in the thousands, and that copy number and polymorphic variation differ across individuals and ethnic groups, the reported null findings should therefore be interpreted with caution (Feusier *et al.*, 2017). In addition, the null associations may partly reflect the use of ICSI, which bypasses natural sperm penetration and selection processes that are present in traditional IVF or natural conception.

Shi *et al.* (2022) investigated associations between mtDNAcn and ART outcomes in 401 men, of whom 126 couples proceeded to IVF or ICSI, and 79 underwent embryo transfer (Shi *et al.*, 2022). Elevated mtDNAcn, quantified by TaqMan-based real-time qPCR using RNase P as the nuclear reference, was statistically associated with poor semen quality (OR 1.10, 95% CI: 1.04, 1.17) and, in unadjusted analyses, with lower embryo fertilization rate (β = −0.63, 95% CI −1.18, −0.08); however, this association was attenuated following adjustment for male and female age, semen quality, ART strategy, and cycle-related variables in quartile analysis. The authors did not report adjusted linear models between mtDNAcn and fertilization rates. No associations between sperm mtDNAcn and other reproductive outcomes were observed, including Day 3 embryo cleavage rate, Day 5 top-quality embryo rate, as well as clinical pregnancy or live birth for the 79 couples with embryo transfers (Shi *et al.*, 2022).

Collectively, the majority of these studies provide convergent evidence that elevated mtDNAcn is associated with impaired fertility outcomes in both natural and assisted conception. The strongest associations are most consistently observed for fertilization success and early embryo quality, whereas associations with clinical pregnancy and live birth appear more variable and likely influenced by additional biological and clinical factors. It is worth noting that mtDNAcn is almost always measured in bulk sperm samples, capturing an ejaculate-wide average that obscures variation at the level of individual sperm. Thus, mtDNAcn differences observed between groups may be driven by a subset of sperm within the sample, a limitation that carries particular relevance in ICSI, where a single spermatozoon is selected for fertilization. Notably, ICSI selection is guided primarily by sperm morphology, and given the consistent association between elevated mtDNAcn and impaired morphology, morphology-based selection may indirectly favor sperm with lower mtDNAcn. Single-cell quantification of mtDNAcn represents an important avenue for future work to better characterize intra-ejaculate heterogeneity and its reproductive implications. Nonetheless, mtDNAcn remains a promising biomarker of early reproductive potential and warrants further investigation in larger prospective, multi-center ART and general population cohorts.

## Interplay between sperm mtDNAcn and sperm nuclear DNA methylation

Given the consistent relationship between elevated mtDNAcn and reduced semen quality across multiple parameters, uncovering additional biological pathways that may mediate these relationships is warranted. One such pathway involves epigenetic regulation of the nuclear genome. Emerging evidence supports a functional interplay between mtDNAcn and nuclear DNA methylation, suggesting a potential role for epigenetic mechanism in regulating mitochondrial dynamics and sperm function in the male germline. This section summarizes current evidence linking sperm mtDNAcn with nuclear DNA methylation and discusses potential mechanistic implications.

In a study of 47 men undergoing fertility evaluation, Oluwayiose *et al.* (2020) assessed sperm mtDNAcn by qPCR and genome-wide DNA methylation using the Illumina Infinium HumanMethylation450K 450K array. They identified 218 differentially methylated regions (DMRs) associated with mtDNAcn (FDR < 0.05), with the vast majority showing increased methylation in men with higher mtDNAcn. Notably, cytosine-phosphate-guanines (CpGs) located near key mitochondrial replication genes, including POLG and TWNK, were among the most strongly associated loci. DNA methylation at imprinted control regions of paternally expressed genes such as *MEST*, *KCNQ1OT1*, *PLAGL1*, and *GRB10* also correlated positively with mtDNAcn. Enrichment analyses revealed that these DMRs were concentrated in regions of nucleosome retained, CpG shores, promoters, and exons. Together, these findings suggest that variation in sperm mtDNAcn may be epigenetically regulated via methylation at loci integral to mitochondrial function and imprinting (Oluwayiose *et al.*, 2020).

Similarly, Tian *et al.* (2014) examined 118 male partners from idiopathic infertile couples and explored the relationship between sperm mtDNAcn and gene-specific promoter methylation. Using real-time PCR and targeted bisulfite sequencing, they evaluated methylation at the imprinted genes *LIT1* and *H19*, as well as *BRDT* and *MTHFR*, which are involved in spermatogenesis. Sperm mtDNAcn was positively correlated with methylation at LIT1 (R = 0.24), BRDT (R = 0.28), and MTHFR (R = 0.21). These associations suggest that dysregulated promoter methylation at key imprinted and germ cell-expressed genes may contribute to abnormal mitochondrial DNA retention during spermatogenesis. Collectively, these studies highlight a potentially coordinated epigenetic–mitochondrial axis in sperm, linking nuclear methylation landscapes with mtDNA content, and implicating roles for both in male fertility potential (Tian *et al.*, 2014).

## Clinical relevance, limitations, and future directions

Elevated mtDNAcn has been consistently associated with poor semen quality, reduced fertilization potential, and prolonged time-to-pregnancy, indicating its potential as a diagnostic marker for subclinical male infertility, particularly in idiopathic cases where standard semen parameters appear normal (Geng *et al.*, 2024; Mai *et al.*, 2024; Vahedi Raad *et al.*, 2024; Arya *et al.*, 2025). Incorporating mtDNAcn into routine diagnostics may enhance the sensitivity of male fertility assessments and provide a more comprehensive view of sperm function (Rosati *et al.*, 2020; Sawant *et al.*, 2025). In ART, mtDNAcn has additionally been proposed as a prognostic marker for embryo viability (Wu *et al.*, 2019b). Taken together, these associations suggest that mtDNAcn may reflect defects in spermatogenesis or mitochondrial clearance processes that compromise early embryonic development (Kavoosi *et al.*, 2024). Accordingly, integration of sperm mtDNAcn assessment into ART workflows could aid in identifying patients who may benefit from alternative clinical strategies or targeted interventions.

Despite its potential, several methodological and biological challenges must be addressed before sperm mtDNAcn can be clinically adopted. Quantification methods, ranging from qPCR and dPCR to next-generation sequencing, differ in sensitivity, normalization, and reference standards (Bagdonaite *et al.*, 2025). Somatic cell contamination is a major concern in sperm mtDNAcn measurement as somatic cells in the ejaculate contribute disproportionately high mitochondrial DNA content. To address this, one study implemented cell type-specific DNA methylation profiling, using DMRs that reliably distinguish sperm from somatic cells, thereby enabling objective identification and exclusion of contaminated samples (Jenkins *et al.*, 2018). A more recent approach extended this strategy by applying supervised statistical modeling trained on sperm and blood methylation data to classify sample purity, allowing contamination to be detected and corrected at the statistical analysis level rather than relying solely on microscopic assessment (Norton *et al.*, 2024). Together, these approaches demonstrate that molecular pre-screening using methylation signatures is essential for ensuring mtDNAcn reflects true sperm mitochondrial content rather than somatic cell contamination. Moreover, mtDNAcn levels may fluctuate due to age, oxidative stress, environmental exposures, and lifestyle factors, raising concerns about temporal stability and individual variability (Vozdova *et al.*, 2022). Without standardized protocols and validated reference ranges, interpretation of mtDNAcn values remains challenging in a clinical setting. Standardization efforts are essential for establishing its utility as a reliable diagnostic tool of male fertility.

From a clinical standpoint, the utility of mtDNAcn may be greatest in cases of idiopathic male infertility, where conventional semen parameters do not fully explain the underlying etiology. Thus, future studies are needed to examine the relationship between mtDNAcn and reproductive outcomes among couples in which men are normospermic. In men with an established etiology, such as varicocele, reductions in mtDNAcn have been observed following varicocelectomy(Gabriel *et al.*, 2012; Arya *et al.*, 2025), indicating that mtDNAcn is responsive to surgical interventions and supporting the notion that testicular temperature or oxidative stress may influence mtDNAcn. Mitochondria-targeted antioxidant therapies such as Coenzyme Q10 and ROS scavengers have shown potential in preserving sperm quality and mitochondrial function under oxidative stress and during cryopreservation, although further studies are needed to examine these therapies in a mtDNAcn-specific manner (Lanconi *et al.*, 2021; Xu *et al.*, 2025). Experimental strategies targeting mitophagy, mitochondrial fusion/fission dynamics, and biogenesis may contribute to restoring mtDNA homeostasis and improving sperm fitness (Tiwari *et al.*, 2022). Furthermore, mtDNA heteroplasmy has been shown to impact fertility by impairing ATP production in early spermatogenesis, resulting in asthenozoospermia, but limited data are available on how elevated heteroplasmic mtDNA in sperm may affect sperm quality (Vertika *et al.*, 2020). Together, these approaches highlight a multifaceted research agenda encompassing therapeutic trials, mechanistic studies, and the development of precision tools to improve sperm quality and reproductive outcomes through via regulation of sperm mtDNAcn.

MtDNAcn may offer additional diagnostic value by serving as a useful marker of treatment response; as demonstrated by Gabriel *et al.* (2012), who showed that microsurgical varicocelectomy significantly reduced sperm mtDNAcn alongside improvements in sperm parameters, and Arya *et al.* (2025), who found that both varicocelectomy and antioxidant supplementation reduced mtDNAcn and oxidative stress in varicocele patients. Targeted application in unexplained cases, combined with efforts to standardize measurement methodologies across laboratories, will be essential before sperm mtDNAcn can be meaningfully incorporated into routine fertility assessment.

## Conclusion

Sperm mtDNAcn represents a potential integrative biomarker that bridges mitochondrial function, genomic integrity, and male fertility potential. Across diverse populations and study designs, elevated sperm mtDNAcn has been consistently associated with impaired semen quality, reduced fertilization success, and prolonged time to pregnancy. Unlike conventional semen parameters, mtDNAcn provides insight into the mitochondrial health of sperm and may capture aspects of sperm fitness not evident from conventional semen analyses. Furthermore, emerging data indicate that sperm mtDNAcn may be epigenetically regulated, linking it to broader genomic and environmental influences on spermatogenesis. Future studies should aim to define normative ranges, clarify causal mechanisms, and explore interventions that modulate mitochondrial DNA content to improve reproductive outcomes. Ultimately, incorporating mtDNAcn into the clinical andrology toolkit may better refine male fertility assessment, inform ART strategies, and support the development of novel therapeutic approaches in reproductive medicine.

## Data Availability

There are no new data associated with this article; no new data were generated or analysed in support of this research.
